# Connecting Soil Organic Carbon and Root Biomass with Land-Use and Vegetation in Temperate Grassland

**DOI:** 10.1155/2014/487563

**Published:** 2014-10-20

**Authors:** Devan Allen McGranahan, Aaron L. Daigh, Jessica J. Veenstra, David M. Engle, James R. Miller, Diane M. Debinski

**Affiliations:** ^1^School of Natural Resource Sciences Range Science Program, North Dakota State University, Fargo, ND 58108-6050, USA; ^2^Natural Sciences, Flagler College, St. Augustine, FL 32085-1027, USA; ^3^Department of Natural Resource Ecology and Management, Oklahoma State University, Stillwater, OK 74078-6013, USA; ^4^Natural Resources and Environmental Sciences, University of Illinois, Urbana, IL 61801, USA; ^5^Department of Ecology, Evolution, and Organismal Ecology, Iowa State University, Ames, IA 50011, USA

## Abstract

Soils contain much of Earth's terrestrial organic carbon but are sensitive to land-use. Rangelands are important to carbon dynamics and are among ecosystems most widely impacted by land-use. While common practices like grazing, fire, and tillage affect soil properties directly related to soil carbon dynamics, their magnitude and direction of change vary among ecosystems and with intensity of disturbance. We describe variability in soil organic carbon (SOC) and root biomass—sampled from 0–170 cm and 0–100 cm, respectively—in terms of soil properties, land-use history, current management, and plant community composition using linear regression and multivariate ordination. Despite consistency in average values of SOC and root biomass between our data and data from rangelands worldwide, broad ranges in root biomass and SOC in our data suggest these variables are affected by other site-specific factors. Pastures with a recent history of severe grazing had reduced root biomass and greater bulk density. Ordination suggests greater exotic species richness is associated with lower root biomass but the relationship was not apparent when an invasive species of management concern was specifically tested. We discuss how unexplained variability in belowground properties can complicate measurement and prediction of ecosystem processes such as carbon sequestration.

## 1. Introduction

Soils constitute the greatest stock of terrestrial organic carbon [1] and soil properties can be affected by land-use and management [2, 3]. Globally, approximately one-quarter of the potential carbon sequestration in soils occurs in rangelands [4]. Rangelands are also one of the most widespread human-impacted biomes on Earth [5], making their role in carbon sequestration sensitive to land-use and climate change [6, 7].

The effects of grazing and fire, specifically, can be varied and opposing. In rangeland, grazing can increase bulk density [8, 9] and has a neutral or negative effect on soil organic carbon (SOC) [10–12]. Likewise, grazing can either increase or decrease root production [13, 14]. Fire increases root growth in tallgrass prairie [13, 15], but across fire-adapted ecosystems the effect of fire on soil carbon varies with severity and temporal scale [16–19]. Temporal scale is especially important to SOC because although SOC can decline over just a few years, SOC accumulation occurs on the scale of decades [20, 21].

Vegetation affects soil carbon stocks by depositing organic matter in the soil. Two major pathways of organic matter input—root tissue and exudates—directly involve plants [22]. Roots contribute to SOC pools through rhizodeposition [23] and the longer residence time of carbon from root tissue than shoot tissue [24]. Abiotic factors can also affect root growth and SOC. Soil clay content has been associated with greater SOC in many soils [25–27]. Bulk density can limit root growth and decrease SOC [28, 29].

Vegetation changes that affect organic matter input deep in the soil profile have an important impact on carbon dynamics. For instance, invasive deep-rooted, warm-season (C_4_) grasses can increase carbon sequestration by increasing organic matter deposition deep in the soil profile [30]. Conversely, the invasion of shallow-rooted, cool-season (C_3_) grasses might reduce soil carbon because cool-season grasses contribute less root tissue than native warm-season grasses deep in the soil profile [31, 32] despite greater root biomass near the soil surface [33].

Carbon dynamics deep in the soil profile are especially important but infrequently studied. Increasing the considered depth from 100 cm to 300 cm increases the global SOC budget by 56% [34]. Carbon deeper in the soil profile is subject to different soil structure, chemistry, and biotic activity, which might contribute to greater carbon sequestration [35].

We studied the effect of grazing history, prescribed fire, and the invasion of an exotic C_3_ grass on root biomass and SOC in rangeland managed with fire and grazing in the tallgrass prairie region of central North America. We describe variability in SOC and root biomass—sampled from 0–170 cm and 0–100 cm, respectively—in terms of soil properties, land-use history, current management, and plant community composition using linear regression and multivariate ordination methods. We expected pastures with a recent history of severe grazing to have less root biomass and lower SOC. We also expected plots with greater abundance of exotic C_3_ invasive species to have less root biomass. Finally, we expected to associate belowground properties with variation in aboveground plant community composition.

## 2. Methods

### 2.1. Study Location and Site History

Our research was conducted in conjunction with ongoing research in the Grand River Grasslands, a 30,000 ha working landscape in Ringgold County, IA, and Harrison County, MO [36]. Study tracts were initially identified as having medium to high potential for prairie conservation and restoration, based on the observed presence of native species indicating parcels of remnant prairie (The Nature Conservancy, unpublished data). When the Grand River Grasslands research project began in 2006, a pretreatment vegetation survey of potentially-remnant tracts confirmed a high incidence of native plant species as well as a range of invasion by nonnative plants [37].

Tracts were identified by historical grazing management. Grazing histories were reconstructed through interviews with current and former managers; four of the tracts were reported ungrazed for at least six years prior to the beginning of the study while five had been grazed by cattle (*Bos taurus*) at high stocking rates (ca. 15 animal unit months/ha) (Table 1) [37, 38]. At the time of this study, tracts were assigned to treatments for the purposes of a fire and grazing experiment that divided the tracts into moderately grazed and ungrazed [39]. We did not expect these recent changes in management to affect belowground soil and root properties (although the effect was tested as part of multivariate analyses, see below). However, because fire has been shown to reduce belowground biomass in tallgrass prairie, for example [15], we did record time-since-fire for each patch for inclusion in our analyses.

### 2.2. Sample Collection and Analysis

As part of ongoing research in these tracts, six modified Whittaker plots per pasture were located with respect to soil series as described by McGranahan et al. [37, 40]. All study tracts were classified to the Gara-Armstrong-Pershing association [41]. Two soil series—Gara loam and Armstrong loam (Fine-loamy, mixed, superactive, mesic Mollic Hapludalf, parent material: glacial till; and Fine, smectitic, mesic Aquertic Hapludalf, parent material: loess over palesol formed in glacial till, respectively [41])—dominated the study tracts, and within each tract three plots were located within each soil series. Slopes spanned three classifications (C, D, and E) and ranged from 8 to 35%.

In June 2010, we located 31, 500 m^2^ modified Whittaker plots (permanently-located vegetation survey plots established in 2006 for estimation of canopy cover [37, 42]) across six tracts in the Grand River Grasslands. Although each tract has six permanent plots, some plots were not accessible by the heavy equipment required for soil sampling. At each plot, we sampled the abundance of tall fescue by recording canopy coverage from 10, 0.5 m^2^ quadrats according to the Daubenmire [43] canopy cover index. We used the mean canopy cover of these 10 quadrats to represent tall fescue abundance in our analyses.

We extracted four adjacent 7.5 cm diameter soil cores from the approximate center of each vegetation plot with a vehicle-mounted hydraulic Giddings probe. Three, 100 cm cores were analyzed for root biomass at 20 cm intervals to determine rooting depth. Twenty-centimeter sections of each core were soaked overnight in a 1% solution of sodium hexametaphosphate (Calgon) [14]. We separated root tissue from soil particles with a sieve and bucket arrangement similar to Lauenroth and Whitman [44]: water was flushed through each soaked core section in a 10 mesh sieve mounted atop a 19L bucket. Mineral material sank in the bucket while root tissue that passed through the 10 mesh sieve floated and was collected in a 40 mesh sieve. Root tissue was collected with tweezers from remaining particles in the sieve and dried for 48 hours at 45°C, with root biomass expressed as mass per unit area [31, 32].

The fourth soil core was sampled to 170 cm and analyzed for soil organic carbon (SOC) at varying depth intervals: 10 cm intervals, 0–60 cm; 20 cm intervals, 60–140 cm, and a 30 cm interval from 140 to 170 cm. To standardize depth intervals when making comparisons with root biomass—which was sampled 0–100 cm in 20 cm increments—we summed 10 cm incremental SOC data from the top 0–60 cm into 20 cm increments. These cores were air-dried and stored unsealed.

Prior to laboratory analysis, the fourth core from each plot was also analyzed for variables that might affect root penetration, including clay content and bulk density in 20 cm increments, depth to argillic and gleyed horizons (which indicate clay accumulation and anoxic conditions, resp.), and depth to an observable plow layer. Based on previous experience with these similar soil series, A. Daigh identified potentially root-limiting thresholds for clay content (27%) and bulk density (1.4 g/cm^3^) and for each core determined the depth at which the root-limiting layer was first observed.

Because our study considered only organic carbon, we tested for and eliminated any inorganic carbon fraction from soil samples. Total carbon and inorganic carbon was determined by the Iowa State University Plant and Soil Analysis Lab, Ames, IA, USA, using the dry combustion and modified pressure calcimeter methods. pH was determined with a glass electrode in a 1 : 1 soil to water suspension. Composite samples from each depth interval were analyzed for total carbon and pH. For samples <7.0 pH, total carbon was assumed to equal organic carbon. Samples ≥7.0 pH were reanalyzed for inorganic carbon, which, when subtracted from total carbon, gives the organic carbon fraction. Prior to submission to the lab, we determined soil bulk density via the soil core method to calculate soil organic carbon on a volumetric rather than gravimetric basis, the standard for reporting and comparing soil carbon data globally, for example [34].

### 2.3. Data Analysis

#### 2.3.1. Belowground Properties and Land-Use

To determine the relationship between soil variables (root mass, SOC, and bulk density) and land-use variables (grazing history, time-since-fire, and tall fescue cover) we constructed linear mixed effect regression (LME) models with the lmer function in the lme4 package (version 1.0-5) for the *R* statistical environment (version 3.0.2) [45, 46]. Response variables included both whole core root biomass (0–100 cm) and surface biomass (0–20 cm); whole core SOC (0–170 cm), surface SOC (0–20 cm), and surface percent SOC (0–10 cm); and surface bulk density (0–20 cm). We also compared total SOC, percent SOC, and root mass in the top 0–20 cm against clay content and bulk density (0–20 cm) and tested for a correlation between bulk density and clay content.

In lieu of *P* values as a measure of statistical significance, we estimated 95% confidence intervals for grazing history and tall fescue cover using the simulation method developed by Nakagawa and Cuthill [47], which compares 1000 simulations of the LME model to empirical response variable data. To test the goodness-of-fit of the mixed-effect regression model we calculated a coefficient of determination (*R* ^2^) with a custom rsquared.lme function following Nakagawa and Schielzeth [48]. The rsquared.lme function extracts variance components from the lme model and calculates marginal *R* ^2^ values that represent the goodness-of-fit for the fixed-effect term.

#### 2.3.2. Multivariate Analysis of Soil, Root, and Vegetation Data

We used ordination to identify patterns among root mass, SOC, and soil properties data. We performed a Principal Components Analysis (PCA) with an unconstrained model using the rda function in the vegan package (version 2.0-7) for the *R* statistical environment [49]. We tested several physical and management-related factors against the ordination using the envfit function in vegan; these variables included tall grazing history, tall fescue cover, current grazing treatment, soil series, and slope of sampled plot.

We also used ordination to test for association between plant community composition and variation in soil and root properties. First, we extracted the principal components (PCs) from the PCA of soil and root data. Together, the PCs represent composite variables that each account for a proportion of variation in the root/soil dataset; as PCs are added the proportion of variation explained by the composite variables accumulates. We sought to include as many PCs as necessary to account for at least 70% of variation in the soil/root data.

Second, we set the PCs as constraints in a Constrained Analysis of Proximities (function capscale in vegan), which performs a constrained ordination based on a user-defined distance metric and is similar to the unconstrained ordination Multi-Dimensional Scaling. For plant community data we used the 2006 pretreatment vegetation survey [37] and used the Canberra distance metric. As applied here, the constrained ordination first describes variation in plant community composition along defined axes—in this case, the composite variables of soil/root data represented by the PCs—then proceeds to explain remaining variation via unconstrained ordination. To determine how useful the soil/root composite variables were in describing variation in plant community composition, we compared an unconstrained ordination of the vegetation data to the constrained ordination model using the anova function.

## 3. Results and Discussion

### 3.1. Relationship between Belowground Properties and Depth

**Figure 1:**
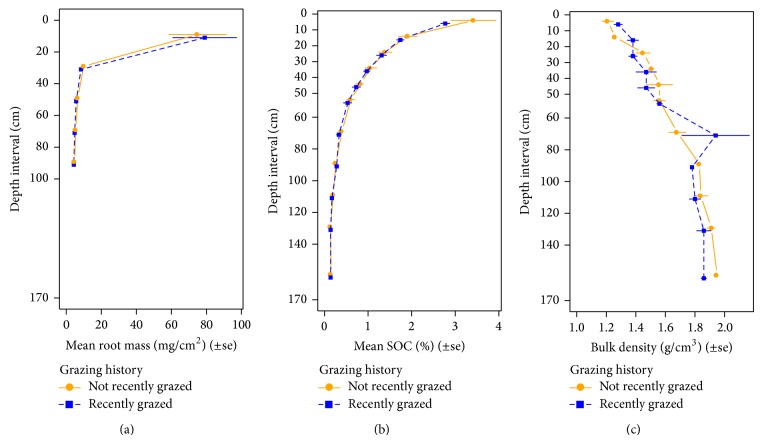
Root biomass, soil organic carbon (SOC), and bulk density for six tallgrass prairie pastures in the Grand River Grasslands of Ringgold County, IA, and Harrison County, MO, USA, plotted by depth (0–170 cm) and grazing history. See Methods for sampling procedures including depth intervals.

Root biomass and SOC decreased rapidly with depth (Figure 1). Bulk density generally increased with depth; wide variation in samples from the 60 to 80 cm increment (Figure 1(c)) reflects a low-density sand lens (likely an isolated/local variation in the glacial till) in a subset of samples. Across all plots, the 0–20 cm increment of the soil profile contained the greatest root biomass and SOC (Table 2). In these soils, sampling to 100 cm increased SOC stock and root biomass by an average of 45% and 40%, respectively, when compared to amounts contained in the top 20 cm of the profile. For SOC, extending the sampling depth to 170 cm increased the estimate of SOC stock in the profile by an average of 63%. Deep sampling clearly increases the amount of organic carbon and root biomass accounted for under these grasslands.

In terms of vertical distribution, root biomass and SOC tended to be concentrated in the upper portion of the soil profile, a pattern consistent with other work. On average, we found 81% of root biomass in the top 40 cm of the soil profile (Table 1), similar to the 83% average for the top 30 cm of temperate grassland worldwide [50]. In another global review, Jobbágy and Jackson [34] report 70% of root biomass concentrated in the 0–20 cm increment and an additional 17% in the 20–40 cm increment, which is congruous with our mean values of 70% and 11%, respectively. Regarding SOC, Jobbágy and Jackson [34] report an average total of 16 kg/m^2^ for the top 200 cm in temperate grasslands worldwide, very near the 17 kg/m^2^ mean reported here. Broken down by depth increments, Jobbágy and Jackson [34] report an average of 42% of SOC distributed in the 0–20 cm increment and 23% in the 20–40 cm increment, again consistent with our values of 37% and 20%, respectively.

### 3.2. Land-Use versus Natural Variation in Belowground Properties

Our data indicate that previous grazing management has affected both belowground and aboveground properties of these grasslands. Pastures with a recent history of severe grazing had lower root biomass in both the top 20 cm and the entire 100 cm profile than other pastures, and greater bulk density (Table 3). These results indicate both biotic and abiotic effects of soil compaction associated with intensive livestock management [11, 13, 51]. That these effects persist at least five years following the cessation of severe grazing highlights the influence of land-use legacies on biophysical properties and ecological pattern and process [52, 53]. Likewise, these recently severely-grazed pastures have different plant community composition, including lower native species richness and a greater abundance of tall fescue [37, 40]. But tall fescue abundance was associated with neither SOC nor root biomass (Table 3). Likewise, current management showed no effect on belowground properties. Specifically, prescribed fire had no association with root biomass or SOC in the top 20 cm of the soil profile (95% CI = −2.6–0.7, *R* ^2^ = 0.05 and 95% CI = −1407–225, *R* ^2^ = 0.06, resp.).

We observed several properties in the upper portion of the soil column that could physically impair root penetration. The average depth of the shallowest root-limiting layer was 12 (±2) cm, and it appears to have an association with reduced root biomass in the top 20 cm of the soil profile (95% CI = 0.01–0.11, *R* ^2^ = 0.13), but not percent SOC (95% CI = −0.02–0.04, *R* ^2^ = 0.01). There was no correlation between bulk density and clay content in the top 20 cm, and neither had an association with SOC or root biomass at the same depth (Table 4), contrary to predictions [29].

Nine plots on four of the six pastures showed evidence of a plow layer, observed at an average depth of 7.7 (±1.5) cm. These data suggest that cultivation had occurred at some point since European settlement of the area in the late 19th century, contrary to our previous efforts to document agricultural histories (interviews with managers and searches through local US Department of Agriculture records) that presented no evidence of tillage. While historical cultivation is not surprising and is in fact expected, it is clear that even land-use activity that occurred long ago and/or briefly in time can leave a long-lasting imprint on soil. But the long-term impact of such activity is less clear: the presence of a plow layer was not associated with differences in bulk density (95% CI = −0.12–0.07, *R* ^2^ = 0.01) or amount of SOC (95% CI = −284–1005, *R* ^2^ = 0.03) in the top 20 cm of soil profiles. For these pastures, it is possible that tillage occurred so long ago that soil has since recovered [20].

### 3.3. Multivariate Analyses of Belowground Properties and Vegetation

**Figure 2:**
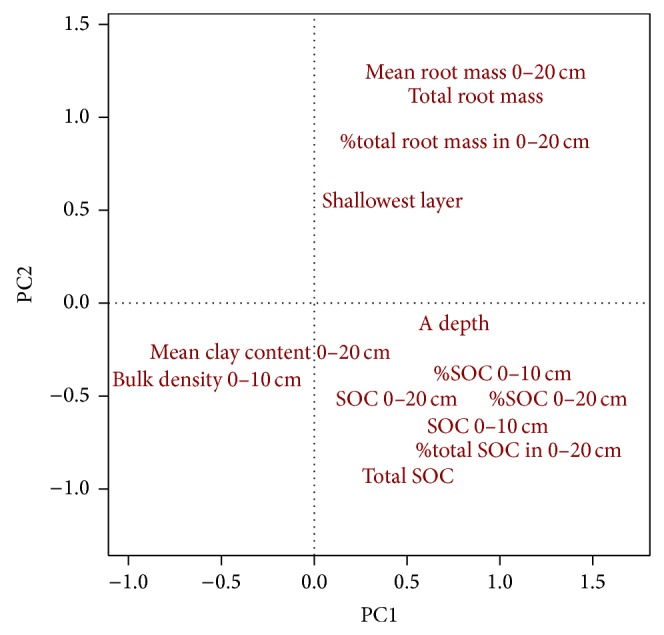
Relationships among 13 belowground properties with respect to first two Principal Components from Principal Components Analysis. For description of plotted text codes and quantified loadings for each variable, see Table 4.

Although the ordination of soil and root data highlighted several patterns among belowground properties, we did not observe patterns that indicate associations between belowground properties, land-use, and vegetation. The PCA revealed three general trends in variation within the soil and root data along which correlated variables clustered (Figure 2): one following root biomass, another SOC, and a third combining clay content and bulk density. Variables related to SOC loaded most heavily along PC1 while variables related to root biomass contributed in greater proportion to PC2 (Table 5). The first three axes of the PCA accounted for 72% of the variation in belowground properties, but there was no association between the PCA and factors that might explain variation in belowground properties, including historical and current grazing, tall fescue abundance, soil series, and slope (*P* > 0.1). We did not expect current management to affect either root biomass or SOC given the low severity and brief time span of the experimental disturbance regime [18, 54].

Not surprisingly—given the concentration of root biomass and SOC near the soil surface (Table 2)—total root biomass (0–100 cm) and total SOC (0–170 cm) each clustered with their respective shallow measures (0–20 cm). These results suggest that unless a complete accounting of the carbon budget is required, shallow sampling is sufficient to characterize root and SOC dynamics through much of the soil column.

An unexpected result of the multivariate analysis is the relationship between root biomass and SOC. Whereas we expected a direct, linear correlation between these two variables, they in fact occur orthogonal to each other in ordination space (Figure 2). Both root biomass and SOC variables appear to have a negative relationship with clay content and bulk density, although no associations were indicated by linear regression models (Table 4). The ordination supports above evidence that deeper root-limiting layers are associated with greater root biomass and suggests that deeper topsoil (A horizon) might tend to have greater root biomass and SOC.

**Figure 3:**
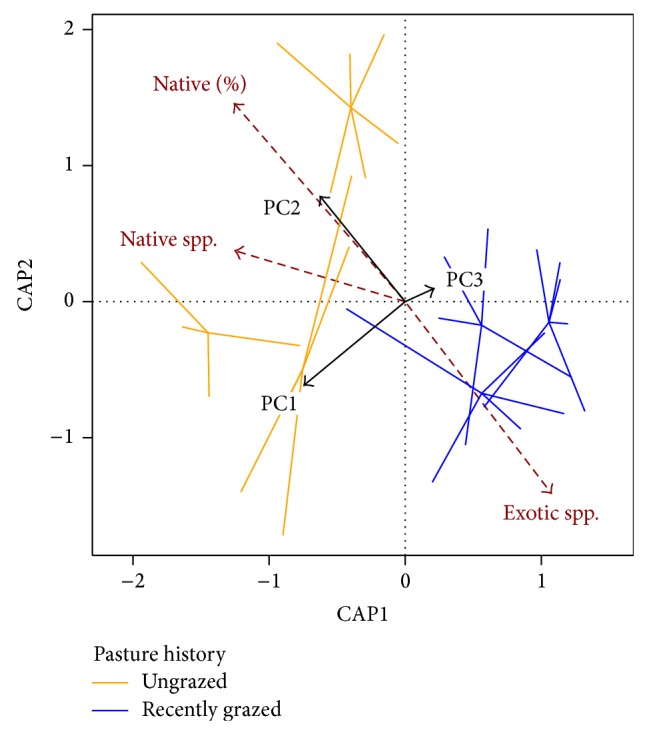
Results of a constrained ordination of plant community composition using composite variables of belowground properties created from soil and root PCA. Spiderplots group sampled modified Whittaker plots by grazing history, thick black arrows show constraining variables, and thin red arrows overlay plant community metadata fitted to the ordination (“Native (%)” = proportion of native species in community, “Native spp.” = native species richness, and “Exotic spp.” = exotic species richness).

There is evidence that plant community composition is associated with variation in belowground properties. PC1 and PC2—composite variables from the PCA ordination of multivariate soil and root data most influenced by 0–10 cm SOC and 0–20 cm root biomass, respectively (Table 5)—were significant terms in the constrained ordination of plant community composition (PC1: *P* = 0.01, PC2: *P* = 0.04). Likewise, the first axis of the constrained ordination was significantly associated with variation in plant community composition (CAP1: *P* = 0.005). There appears to be an association between PC2 and exotic species richness in ordination space (Figure 3); although PC2 is the axis of secondary variation in the PCA, it is also the axis most influenced by root biomass. Thus, these data might suggest a connection between greater exotic species richness and reduced root biomass, which supports a pattern seen elsewhere in tallgrass prairie documenting greater root biomass among native species versus exotic species [32]. But the three constraining axes explained just 13% of variation in the ordination of vegetation data, leaving substantial amount of variation in plant community composition unexplained by belowground properties. As shown elsewhere, grazing history, tall fescue abundance, and the ratio of native to exotic species strongly influence plant community composition in these grasslands [37, 39].

### 3.4. Land-Use, Variability, and Ecosystem Processes

Given that sampling deeper in the soil profile makes important contributions to the global carbon cycle [34], we sampled soil organic carbon (SOC) and root biomass to 170 cm and 100 cm, respectively, in temperate rangeland soils. Although on one hand such deep sampling did increase the amount of soil organic carbon we can account for in this system, on the other hand our ability to relate these data to ecosystem-level processes like carbon sequestration is limited by (i) the high degree of variability we observed in belowground properties at shallow soil layers and (ii) the lack of pattern between variability in belowground properties and known land-uses and plant composition.

Most relevant to carbon budgets, we did not observe any relationship between vegetation and SOC or root biomass at any level of the soil profile, let alone at depth. Although the majority of organic matter is found in the upper strata of the soil, carbon deep in the soil profile might have a disproportionately greater influence on the long-term carbon cycle if deep carbon has a longer residence time than carbon higher in the profile, for example [55]. Other studies indicate exotic plants can affect the vertical distribution of root biomass in native communities [30–32], but greater root biomass in native grass versus tall fescue stands does not necessarily lead to increased SOC [56]. Likewise, our results do not suggest that tall fescue, specifically, affects root biomass or SOC in these rangelands at either the fine or broad temporal scales considered here, despite indications of a community-level association between exotic species richness and reduced root biomass (Figure 3). If one were to consider the gradient of invasion/floristic degradation across the study tracts as a space-for-time substitution, for example [57], one could draw two conclusions: either invasive species might not reduce the carbon sequestration potential of these rangelands or the restoration of native plant dominance does not seem likely to increase carbon sequestration potential of invaded/degraded rangeland in the foreseeable future.

## 4. Conclusion

Although sampling deep into the soil profile substantially increased estimated stocks of SOC and root biomass in these rangelands, observed dynamics between soil properties, management, and plant communities appear restricted to the upper 20 cm of the soil profile. These results corroborate two important themes in soil carbon research. First, increasing the sampling depth contributes to substantially greater soil organic carbon (SOC) stocks in temperate grassland soils. Second, vegetation, land-use management, and soil properties interact to affect soil carbon and root biomass stocks, but this relationship is not necessarily straightforward. Together, these results support the claim that rangeland soils are important carbon pools but also suggest it is unlikely that rangeland plant communities can be effectively categorized by their carbon sequestration potential without considering biotic and abiotic factors, even within climate zones and regions.

## Figures and Tables

**Table 1:** Summary of historical (2000–2006) and 2010 grazing management information and tall fescue abundance (as percent canopy cover) for six pastures in the Grand River Grasslands of Ringgold County, IA, and Harrison County, MO, USA. Severe grazing refers to approximately 15 animal unit months/ha [37].

Pasture	Grazing		Tall fescue canopy cover (%)		
	Historical	Current	Minimum	Mean (±se)	Maximum
Lee Trail	Not recently grazed	Moderately grazed	13	35 (±8)	61
Pawnee	Not recently grazed	Ungrazed	0	<1	<1
Pyland North	Recently severely grazed	Moderately grazed	38	59 (±7)	75
Pyland South	Recently severely grazed	Moderately grazed	20	37 (±5)	53
Pyland West	Recently severely grazed	Moderately grazed	17	50 (±8)	63
Ringgold North	Not recently grazed	Ungrazed	0	<1	1

**Table 2:** Descriptive statistics for soil organic carbon (SOC) and root biomass from 31 vegetation plots across five study tracts in the Grand River Grasslands of Ringgold County, IA, and Harrison County, MO, USA.

Variable	Minimum	Mean	Maximum
Total SOC in 170 cm profile (kg/m^2^)	11.4	16.8	27.4
Percent of total SOC in top 20 cm	21.5%	37.2%	51.4%
Percent of total SOC in top 40 cm	28.8%	56.9%	71.5%
Percent of total SOC in top 100 cm	38.0%	81.9%	96.9%
Total root biomass in 100 cm profile (mg/cm^2^)	53.6	102.0	212.4
Percent of total root biomass in top 20 cm	33.1%	69.8%	96.5%
Percent of total root biomass in top 40 cm	52.8%	80.5%	98.0%

**Table 3:** Results of six multiple linear mixed effect regression models each comparing response variables against tall fescue abundance and grazing history as independent variables (fixed effects). Lower and upper bounds define simulated 95% confidence intervals while *R* ^2^ reports goodness-of-fit for multiple fixed effects.

Response variable	Tall fescue		Grazing history		*R* ^2^
	Lower	Upper	Lower	Upper	
Soil organic carbon					
0–20 cm	−2.9	20.6	−927	316	0.07
0–100 cm	−58	115	−5339	3307	0.01
% 0–10 cm	−0.02	0.02	−1.68	0.51	0.10
Root mass					
0–20 cm	−0.01	0.06	−4.1	−0.9	0.24
0–100 cm	−0.01	0.18	−13.7	−3.6	0.28
Bulk density					
0–20 cm	−0.002	0.002	0.02	0.2	0.22

**Table 4:** Results of four multiple linear mixed effect regression models each comparing response variables against bulk density and clay content as independent variables (fixed effects). Lower and upper bounds define simulated 95% confidence intervals while *R* ^2^ reports goodness-of-fit for multiple fixed effects.

Response variable	Bulk density		Clay content		*R* ^2^
	Lower	Upper	Lower	Upper	
Soil organic carbon					
SOC 0–20 cm	−2052	1857	−33.5	15.5	0.01
% 0–10 cm	−4.8	0.7	−0.03	0.04	0.07

Root mass (0–20 cm)	−10.8	2.2	−0.09	0.06	0.06

Bulk density (0–20 cm)			−0.006	0.002	0.03

**Table 5:** Loadings along first three Principal Components (PC1, PC2, and PC3) for 13 belowground properties measured from six pastures in the Grand River Grasslands of Ringgold County, IA, and Harrison County, MO, USA. Plotting code refers to plotted text in Figure 2.

Variable type	Plotting code	Meaning	PC1	PC2	PC3
Soil organic carbon (SOC)	Total SOC	Total mass of SOC in 0–100 cm column	0.76	−0.54	−0.5
	SOC 0–10 cm	SOC mass in 0–10 cm	0.98	−0.33	0.63
	SOC 0–20 cm	SOC mass in 0–20 cm	0.79	−0.37	−0.68
	% total SOC 0–10 cm	Proportion of total SOC mass in 0–10 cm	0.98	−0.31	0.63
	% total SOC 0–20 cm	Proportion of total SOC mass in 0–20 cm	1.13	−0.43	0.13
	% SOC 0–20 cm	% SOC, 0–20 cm	1.12	−0.35	0.12

Root mass	Total root mass	Total root biomass in 0–100 cm column	0.73	0.9	0.07
	Mean root mass 20 cm	Root biomass in 0–20 cm	0.77	0.91	0.07
	% total root mass in 0–20 cm	Proportion of total root biomass in 0–20 cm	0.7	0.81	0.13

Root limitations	Mean clay content 0–20 cm	Average clay content of 0–10 and 11–20 cm intervals	−0.11	−0.22	0.51
	Bulk density 0–10 cm	Bulk density 0–10 cm	−0.43	−0.34	0.21
	Shallowest layer	Shallowest depth (cm) of a soil property expected to limit root penetration^1^	0.38	0.47	−0.29
	A depth	Depth of a horizon (topsoil)	0.81	−0.09	−0.66

^1^Variables used to determine limitation to rooting depth: clay content = 27%, bulk density = 1.4, observation of argillic or gleyed horizon, or a plow layer.

## References

[1] Batjes N. H. (1996). Total carbon and nitrogen in the soils of the world. *European Journal of Soil Science*.

[2] da Silva A. P., Kay B. D., Perfect E. (1997). Management versus inherent soil properties effects on bulk density and relative compaction. *Soil and Tillage Research*.

[3] McCulley R. L., Burke I. C., Nelson J. A., Lauenroth W. K., Knapp A. K., Kelly E. F. (2005). Regional patterns in carbon cycling across the Great Plains of North America. *Ecosystems*.

[4] Follett R. F., Reed D. A. (2010). Soil carbon sequestration in grazing lands: societal benefits and policy implications. *Rangeland Ecology and Management*.

[5] Ellis E. C., Ramankutty N. (2008). Putting people in the map: anthropogenic biomes of the world. *Frontiers in Ecology and the Environment*.

[6] Conant R. T., Paustian K. (2002). Potential soil carbon sequestration in overgrazed grassland ecosystems. *Global Biogeochemical Cycles*.

[7] Dean C., Wardell-Johnson G. W., Harper R. J. (2012). Carbon management of commercial rangelands in Australia: major pools and fluxes. *Agriculture, Ecosystems & Environment*.

[8] Manley J. T., Schuman G. E., Reeder J. D., Hart R. H. (1995). Rangeland soil carbon and nitrogen responses to grazing. *Journal of Soil and Water Conservation*.

[9] Pineiro G., Paruelo J. M., Oesterheld M., Jobbágy E. G. (2010). Pathways of grazing effects on soil organic carbon and nitrogen. *Rangeland Ecology and Management*.

[10] Bagchi S., Ritchie M. E. (2010). Introduced grazers can restrict potential soil carbon sequestration through impacts on plant community composition. *Ecology Letters*.

[11] He N. P., Zhang Y. H., Yu Q., Chen Q. S., Pan Q. M., Zhang G. M., Han X. G. (2011). Grazing intensity impacts soil carbon and nitrogen storage of continental steppe. *Ecosphere*.

[12] Medina-Roldán E., Paz-Ferreiro J., Bardgett R. D. (2012). Grazing exclusion affects soil and plant communities, but has no impact on soil carbon storage in an upland grassland. *Agriculture, Ecosystems and Environment*.

[13] Johnson L. C., Matchett J. R. (2001). Fire and grazing regulate belowground processes in tallgrass prairie. *Ecology*.

[14] Pucheta E., Bonamici I., Cabido M., Díaz S. (2004). Below-ground biomass and productivity of a grazed site and a neighbouring ungrazed exclosure in a grassland in central Argentina. *Austral Ecology*.

[15] Limb R. F., Fuhlendorf S. D., Engle D. M., Kerby J. D. (2011). Growing-season disturbance in tallgrass prairie: evaluating fire and grazing on Schizachyrium scoparium. *Rangeland Ecology and Management*.

[16] Neary D. G., Klopatek C. C., DeBano L. F., Ffolliott P. F. (1999). Fire effects on belowground sustainability: a review and synthesis. *Forest Ecology and Management*.

[17] Bird M. I., Veenendaal E. M., Moyo C., Lloyd J., Frost P. (2000). Effect of fire and soil texture on soil carbon in a sub-humid savanna (Matopos, Zimbabwe). *Geoderma*.

[18] Fynn R. W. S., Haynes R. J., O'Connor T. G. (2003). Burning causes long-term changes in soil organic matter content of a South African grassland. *Soil Biology and Biochemistry*.

[19] Knicker H. (2007). How does fire affect the nature and stability of soil organic nitrogen and carbon? A review. *Biogeochemistry*.

[20] Baer S. G., Kitchen D. J., Blair J. M., Rice C. W. (2002). Changes in ecosystem structure and function along a chronosequence of restored grasslands. *Ecological Applications*.

[21] Baer S. G., Meyer C. K., Bach E. M., Klopf R. P., Six J. (2010). Contrasting ecosystem recovery on two soil textures: implications for carbon mitigation and grassland conservation. *Ecosphere*.

[22] Rumpel C., Kögel-Knabner I. (2011). Deep soil organic matter—a key but poorly understood component of terrestrial C cycle. *Plant and Soil*.

[23] Wilts A. R., Reicosky D. C., Allmaras R. R., Clapp C. E. (2004). Long-term corn residue effects: harvest alternatives, soil carbon turnover, and root-derived carbon. *Soil Science Society of America Journal*.

[24] Rasse D. P., Rumpel C., Dignac M.-F. (2005). Is soil carbon mostly root carbon? Mechanisms for a specific stabilisation. *Plant and Soil*.

[25] Sollins P., Homann P., Caldwell B. A. (1996). Stabilization and destabilization of soil organic matter: mechanisms and controls. *Geoderma*.

[26] Alvarez R., Lavado R. S. (1998). Climate, organic matter and clay content relationships in the Pampa and Chaco soils, Argentina. *Geoderma*.

[27] Leifeld J., Bassin S., Fuhrer J. (2005). Carbon stocks in Swiss agricultural soils predicted by land-use, soil characteristics, and altitude. *Agriculture, Ecosystems and Environment*.

[28] Lal R., Kimble J. M. (2001). Importance of soil bulk density and methods of its importance. *Assessment Methods for Soil Carbon*.

[29] Brye K. R., West C. P., Gbur E. E. (2004). Soil quality differences under native tallgrass prairie across a climosequence in Arkansas. *The American Midland Naturalist*.

[30] Fisher M. J., Rao I. M., Ayarza M. A., Lascano C. E., Sanz J. I., Thomas R. J., Vera R. R. (1994). Carbon storage by introduced deep-rooted grasses in the South American savannas. *Nature*.

[31] Tufekcioglu A., Raich J. W., Isenhart T. M., Schultz R. C. (1998). Fine root dynamics, coarse root biomass, root distribution, and soil respiration in a multispecies riparian buffer in Central Iowa, USA. *Agroforestry Systems*.

[32] Wilsey B. J., Polley H. W. (2006). Aboveground productivity and root-shoot allocation differ between native and introduced grass species. *Oecologia*.

[33] Fink K. A., Wilson S. D. (2011). Bromus inermis invasion of a native grassland: diversity and resource reduction. *Botany*.

[34] Jobbágy E. G., Jackson R. B. (2000). The vertical distribution of soil organic carbon and its relation to climate and vegetation. *Ecological Applications*.

[35] Blanco-Canqui H., Lal R. (2004). Mechanisms of carbon sequestration in soil aggregates. *Critical Reviews in Plant Sciences*.

[36] Miller J. R., Morton L. W., Engle D. M., Debinski D. M., Harr R. N. (2012). Nature reserves as catalysts for landscape change. *Frontiers in Ecology and the Environment*.

[37] McGranahan D., Engle D., Fuhlendorf S., Miller J., Debinski D. (2013). Multivariate analysis of rangeland vegetation and soil organic carbon describes degradation, informs restoration and conservation. *Land*.

[38] Mcgranahan D. A., Engle D. M., Fuhlendorf S. D., Winter S. J., Miller J. R., Debinski D. M. (2012). Spatial heterogeneity across five rangelands managed with pyric-herbivory. *Journal of Applied Ecology*.

[39] Moranz R. A., Debinski D. M., McGranahan D. A., Engle D. M., Miller J. R. (2012). Untangling the effects of fire, grazing, and land-use legacies on grassland butterfly communities. *Biodiversity and Conservation*.

[40] McGranahan D. A., Engle D. M., Wilsey B. J., Fuhlendorf S. D., Miller J. R., Debinski D. M. (2012). Grazing and an invasive grass confound spatial pattern of exotic and native grassland plant species richness. *Basic and Applied Ecology*.

[41] USDA-NRCS Web Soil Survey data for Ringgold County, Iowa. Natural Resource Conservation Service, United States Department of Agriculture. http://websoilsurvey.nrcs.usda.gov.

[42] Stohlgren T. J., Bull K. A., Otsuki Y. (1998). Comparison of rangeland vegetation sampling techniques in the Central Grasslands. *Journal of Range Management*.

[43] Daubenmire R. (1959). A canopy-coverage method of vegetational analysis. *Northwest Science*.

[44] Lauenroth W. K., Whitman W. C. (1971). A rapid method for washing roots. *Journal of Range Management*.

[45] Bates D., Maechler M., Bolker B., Walker S. lme4: Linear mixed-effects models using Eigen and S4. http://cran.r-project.org/web/packages/lme4/index.html.

[46] R Development Core Team (2013). *R: A Language and Environment for Statistical Computing*.

[47] Nakagawa S., Cuthill I. C. (2007). Effect size, confidence interval and statistical significance: a practical guide for biologists. *Biological Reviews*.

[48] Nakagawa S., Schielzeth H. (2013). A general and simple method for obtaining $R^{2}$ from generalized linear mixed-effects models. *Methods in Ecology and Evolution*.

[49] Oksanen J., Blanchet F. G., Kindt R., Legendre P., O'Hara R. G., Simpson G. L., Solymos P., Stevens M. H. H., Wagner H., Minchin P. R. vegan: Community ecology package. http://cran.r-project.org/web/packages/vegan/index.html.

[50] Jackson R. B., Canadell J., Ehleringer J. R., Mooney H. A., Sala O. E., Schulze E. D. (1996). A global analysis of root distributions for terrestrial biomes. *Oecologia*.

[51] Savadogo P., Sawadogo L., Tiveau D. (2007). Effects of grazing intensity and prescribed fire on soil physical and hydrological properties and pasture yield in the savanna woodlands of Burkina Faso. *Agriculture, Ecosystems and Environment*.

[52] Foster D., Swanson F., Aber J., Burke I., Brokaw N., Tilman D., Knapp A. (2003). The importance of land-use legacies to ecology and conservation. *BioScience*.

[53] Davies K. W., Svejcar T. J., Bates J. D. (2009). Interaction of historical and nonhistorical disturbances maintains native plant communities. *Ecological Applications*.

[54] McGranahan D. A., Engle D. M., Fuhlendorf S. D., Miller J. R., Debinski D. M. (2012). An invasive cool-season grass complicates prescribed fire management in a native warm-season grassland. *Natural Areas Journal*.

[55] Trumbore S. (2000). Age of soil organic matter and soil respiration: radiocarbon constraints on belowground C dynamics. *Ecological Applications*.

[56] Garten C. T., Wullschleger S. D. (1999). Soil carbon inventories under a bioenergy crop (Switchgrass): measurement limitations. *Journal of Environmental Quality*.

[57] Pickett S. T. (1989). Space-for-time substitution as an alternative to long-term studies. *Long-Term Studies in Ecology: Approaches and Alternatives*.

